# Preparation and preliminary evaluation of a tritium-labeled allosteric P2X4 receptor antagonist

**DOI:** 10.1007/s11302-024-10005-2

**Published:** 2024-05-25

**Authors:** Jessica Nagel, Olli Törmäkangas, Katja Kuokkanen, Ali El-Tayeb, Josef Messinger, Aliaa Abdelrahman, Christiane Bous, Anke C. Schiedel, Christa E. Müller

**Affiliations:** 1 PharmaCenter Bonn, Pharmaceutical Institute, University of Bonn, Pharmaceutical & Medicinal Chemistry, An der Immenburg 4, Bonn, 53121 Germany; 2 Orion Pharma, Orion Corporation, Tengströminkatu 8, FI-20360 Turku, and Orionintie 1A, Espoo, FI- 02200 Finland

**Keywords:** Antagonist, Ligand-gated ion channel, Negative allosteric modulator, P2X4 receptor, Radioligand

## Abstract

P2X4 receptors are ATP-gated cation channels that were proposed as novel drug targets due to their role in inflammation and neuropathic pain. Only few potent and selective P2X4 receptor antagonists have been described to date. Labeled tool compounds suitable for P2X4 receptor binding studies are lacking. Here, we present a novel allosteric P2X4 receptor antagonist possessing high potency in the low nanomolar range. We describe its tritium-labeling resulting in the P2X4-selective radiotracer [^3^H]PSB-OR-2020 with high specific activity (45 Ci/mmol; 1.67 TBq/mmol). A radioligand binding assay was developed using human embryonic kidney (HEK293) cell membranes recombinantly expressing the human P2X4 receptor. Competition binding studies with structurally diverse P2X4 receptor antagonists revealed different allosteric binding sites indicating that the new class of P2X4 receptor antagonists, to which PSB-OR-2020 belongs, interacts with an unprecedented allosteric site. [^3^H]PSB-OR-2020 may become a useful tool for research on P2X4 receptors and for promoting drug development.

**Supplementary Information:**

The online version contains supplementary material available at 10.1007/s11302-024-10005-2.

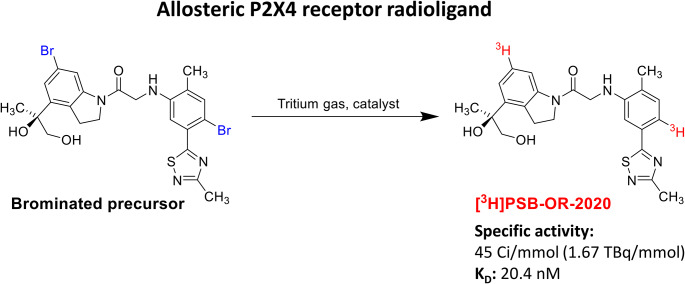

## Introduction

P2X4 receptors belong to the P2X receptor family of ATP-activated ionotropic membrane receptors comprising seven receptor subunits, P2X1-P2X7 [1]. Crystallization and cryogenic electron microscopy (cryo-EM) structures of the zebrafish P2X4 receptor confirmed its previously proposed homotrimeric structure [2–5]. Each protein subunit is composed of two transmembrane domains (TM1 and TM2) and a large extracellular loop [2, 3]. The crystal structure of the zebrafish P2X4 receptor revealed the location of three ATP-binding sites in the extracellular domains, one in each subunit [2, 3]. Binding of the physiological agonist ATP induces receptor subunit rearrangement and ion channel opening for Na^+^, Ca^2+^ and K^+^ ions [3, 6]. In addition to homotrimeric receptors, heterotrimeric receptors exist, e.g. P2X2/3 receptors [1, 4, 7].

P2X4 receptors are expressed in the central and peripheral nervous system, especially in astrocytes, neurons, microglia and endothelial cells [1, 8]. A number of studies have shown that P2X4 receptors are involved in neuropathologies [9]; they appear to play a central role in neuropathic pain [10]. After peripheral nerve injury, microglial cells are activated, and P2X4 receptor expression is strongly upregulated [11–15]. This results in the release of brain-derived neurotrophic factor (BDNF) which initiates a cascade of neuroinflammatory signals resulting in pain and hypersensitivity. The pharmacological blockade of P2X4 receptors was reported to reverse tactile allodynia, a prominent symptom of neuropathic pain [11–14, 16]. P2X4 receptor blockade might also be beneficial for the treatment of other types of chronic pain, e.g. cancer pain [17], inflammatory pain [18, 19], and visceral pain [20]. An upregulation of P2X4 receptors in reactive microglia or in neurons, respectively, was also observed in Alzheimer’s [21] and Parkinson’s disease [22], amyotrophic lateral sclerosis (ALS) [23], epilepsy [24], multiple sclerosis [25] and stroke [26, 27]. The role of P2X4 receptors in all of these neuroinflammatory processes suggests that P2X4 receptor antagonists might be effective drugs for the treatment of associated diseases.

Besides their crucial role in neuroinflammatory diseases, P2X4 receptors have emerged as potential targets for the treatment of cancer, including breast [28], prostate [29], gastric [30], colon [31] and renal cancer [32]. Recently, P2X4 receptors have been reported to play an important role in the fine-tuning of the basal activity and the initial activation of CD4^+^ T cells [33], indicating that the receptors are involved in immune responses to pathogens and cancer cells.

**Fig. 1:**
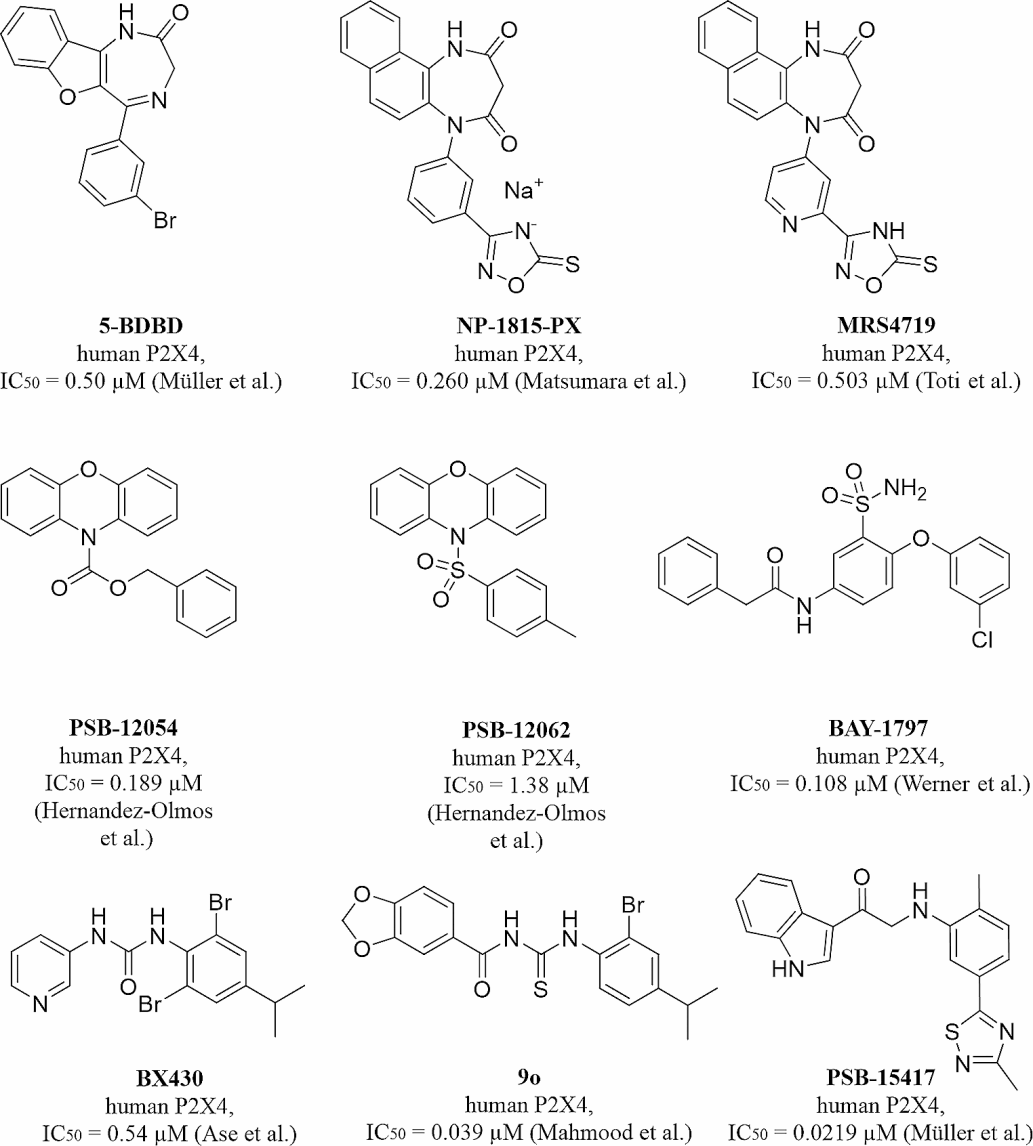
P2X4 receptor antagonists with inhibitory potencies (IC_50_) at the human P2X4 receptor. Müller et al. [36]; Matsumara et al. [37]; Toti et al. [38]; Hernandez-Olmos et al. [39]; Werner et al. [40]; Ase et al. [41]; Mahmood et al. [42]

The discovery of drug-like competitive P2X4 receptor antagonists is hampered by the polar ATP binding site lined with basic amino acid residues [3, 4]. However, several allosteric modulators have been identified and in some cases optimized [34–36]. P2X4 receptor antagonists include benzodiazepine derivatives, e.g. 5-BDBD and derived compounds, e.g. NP-1815-PX [37], and its analog MRS4719 [38], phenoxazine derivatives, e.g. PSB-12054 and PSB-12062 [39], sulfonamide derivatives, e.g. BAY-1797 [40], and the urea derivative BX430 and its analogs, e.g. compound 9o (Fig. 1) [41, 42].

PSB-15417 was developed as a potent, highly selective and brain-permeable allosteric P2X4 antagonist by our group showing efficacy in an animal model of neuropathic pain [14, 36]. NC-2600 (structure undisclosed) was the first P2X4 receptor antagonist to enter clinical trials (phase I) [43]. The effects of NP-1815-PX and NC-2600 were evaluated in a murine model of colitis and found to improve several inflammatory parameters [44]. MRS4719 showed neuroprotective and neuro-rehabilitative effects in a murine ischemic stroke model [38].

Various assays have been developed for studying the potency of P2X receptor ligands. These include calcium influx assays [45], patch-clamp measurements [46], and radioligand binding assays [47, 48]. Radioligands are powerful tools in pharmacology and drug discovery for measuring direct ligand-protein interactions, determining affinities and binding kinetics, including those of unlabeled compounds by performing competition assays. Moreover, radioligands are used for receptor imaging in vitro and in vivo, for autoradiography, scintigraphy (SPECT, single photon emission computer tomography) and positron emission tomography (PET) studies. The orthosteric binding site of the P2X4 receptor can be labeled by the agonist radioligand [^35^S]ATPγS, an ATP analog with higher metabolic stability than ATP [48, 49].

Potent and selective antagonist radioligands for the labeling of allosteric P2X receptor binding sites have so far only been described for P2X3 and P2X7 receptors [47, 50]. Attempts to develop PET radioligands for the P2X4 receptor were described, based on 5-BDBD, which was radiolabeled with ^11^C, ^18^F, or ^76^Br. However, in vitro binding studies with these radiolabeled 5-BDBD analogs have not been successful [51]. This is not surprising given the moderate potency and low water-solubility of 5-BDBD (Fig. 1).

Here, we present the development of a potent, selective allosteric antagonist radioligand for the P2X4 receptor designated [^3^H]PSB-OR-2020. This radiotracer has allowed the development of the first antagonist radioligand binding assay for the human P2X4 receptor.

## Results

**Fig. 2:**
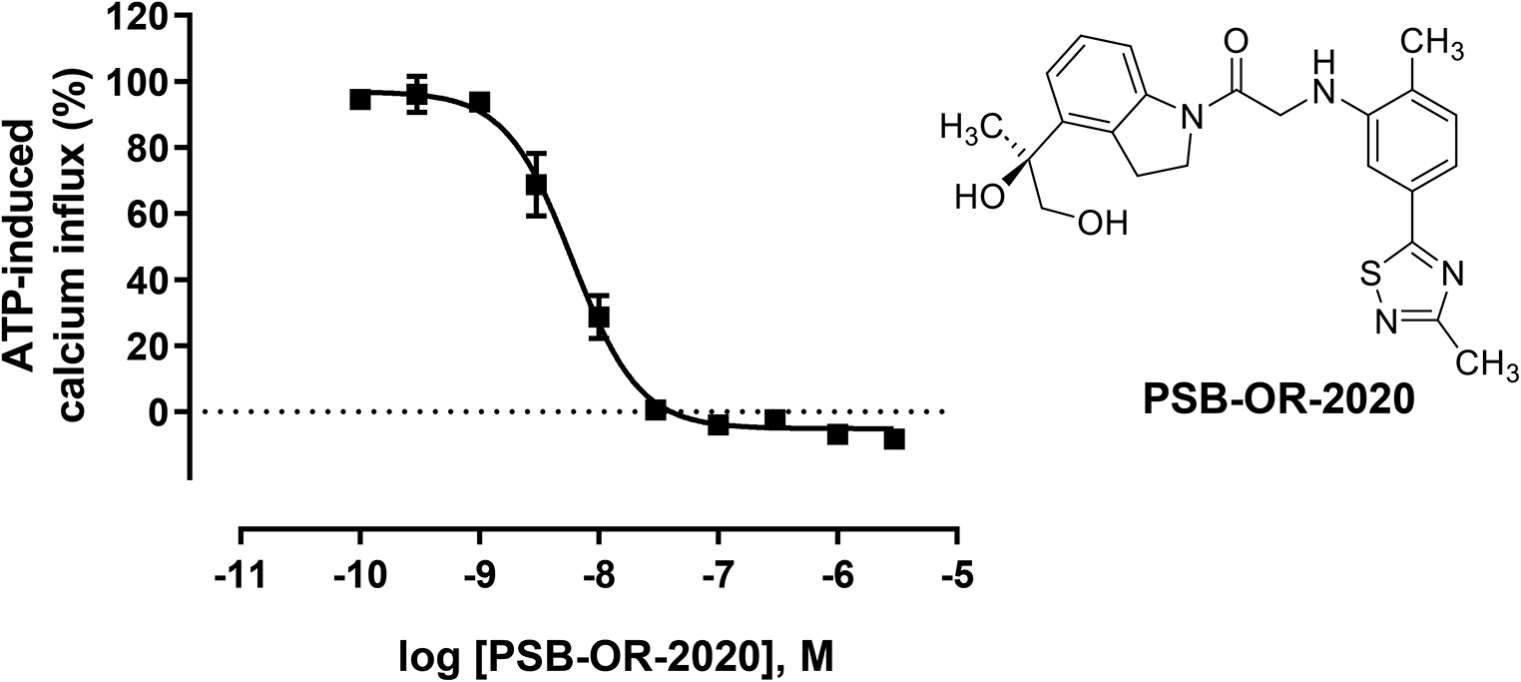
Concentration-dependent inhibition of ATP-induced Ca^2+^ influx by PSB-OR-2020 determined in 1321N1 astrocytoma cells stably transfected with the human P2X4 receptor. An EC_80_ of ATP was applied (300 nM) after preincubation of the cells with various concentrations of PSB-OR-2020. Data points represent means ± SEM of four independent experiments performed in duplicates. IC_50_ value: 6.32 ± 1.52 nM

In the search for novel scaffolds with P2X4 receptor-antagonistic activity, we had previously established assays allowing high throughput screening [49]. This led to the discovery of indolylcarboxymethylaniline derivatives which were subsequently optimized resulting in highly potent P2X4 receptor antagonists, e.g., PSB-15417, with IC_50_ values in the low nanomolar range determined in calcium influx assays (patent application filed in December 2022). Based on the discovery of this new scaffold, we developed a series of related dihydroindole analogs and selected one of the most potent antagonists of this series, (*S*)-1-(4-(1,2-dihydroxypropane-2yl)indolin-1-yl)-2-((2-methyl-5-(3-methyl-1,2,4-thiadiazol-5-yl)phenyl)amino)ethane-1-one, designated PSB-OR-2020, for the preparation of a radioligand (see Fig. 2). The selection was also based on sufficient polarity of the compound to avoid high non-specific binding.

### Characterization of the unlabeled P2X4 receptor antagonist PSB-OR-2020 in calcium influx assays

The unlabeled PSB-OR-2020 was characterized as a highly potent and selective P2X4 receptor antagonist. Calcium influx assays using 1321N1 astrocytoma cells that stably expressed the human P2X4 receptor were performed. Concentration-dependent inhibition of ATP-induced calcium influx was observed. The employed ATP concentration corresponded to its EC_80_ value (300 nM), and an IC_50_ value of 6.32 ± 1.52 nM was determined for the antagonist PSB-OR-2020 (see Fig. 2).

The selectivity of PSB-OR-2020 versus other P2X receptor subtypes was determined in the same type of assay using 1321N1 astrocytoma cells stably expressing the respective human P2X receptor subtype (see Table 1).

**Table 1:** Potency of PSB-OR-2020 as antagonist of human P2X receptor subtypes

P2X receptor subtype	IC_50_ ± SEM (or percent inhibition at indicated concentration)^a^
Human P2X4	6.32 ± 1.52 nM
Human P2X1	> 10,000 nM (9%)
Human P2X2	> 10,000 nM (6%)
Human P2X3	2,030 ± 480 nM
Human P2X7	> 10,000 nM (0%)^b^

^a^Inhibition of ATP-induced signal (EC_80_ concentration: P2X4, 300 nM; P2X1, 100 nM; P2X2, 1,000 nM; P2X3, 100 nM) in the presence of PSB-OR-2020 (10 µM) was determined in 2–3 independent experiments performed in duplicates. Data points represent means ± SEM

^b^For activation of the human P2X7 receptor, the agonist 2’(3’)-O-(4-benzoylbenzoyl)adenosine-5’-triphosphate (Bz-ATP) was used (EC_80_ concentration, 6,000 nM)

At the other human P2X receptor subtypes, P2X1, P2X2, P2X3, and P2X7, inhibition of the agonist-induced signal by a high concentration of PSB-OR-2020 (10 µM) was well below 50%, except for the P2X3 receptor. Thus, a concentration-inhibition curve was determined, resulting in an IC_50_ value of 2,030 ± 480 nM at the P2X3 receptor. This value is about 300-fold higher than the compound’s IC_50_ value at the P2X4 receptor. Thus, PSB-OR-2020 is not only a potent, but also a highly subtype-selective P2X4 receptor antagonist.

## Radiolabeling

**Fig. 3:**
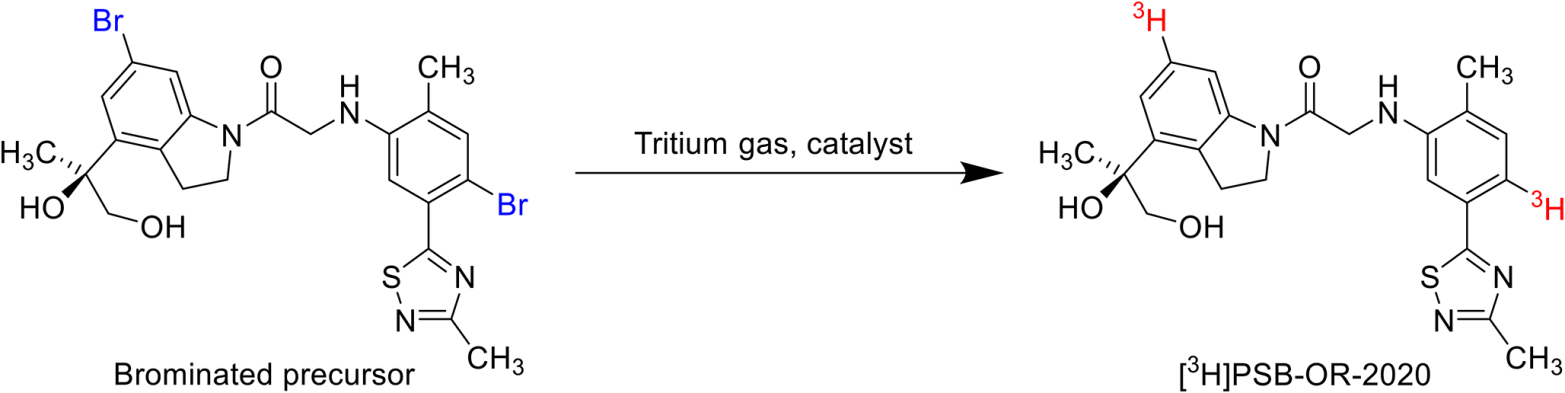
Synthesis of [^3^H]PSB-OR-2020 by catalytic hydrogenation with tritium gas

Having a P2X4 receptor antagonist with low nanomolar potency and high selectivity in hand, we designed a precursor molecule that would be suitable for catalytic hydrogenation with tritium gas (^3^H_2_) to obtain PSB-OR-2020 in ^3^H-labeled form. Thus, we introduced two Br atoms, one on each aromatic ring of the scaffold, which was expected to result in sufficiently high specific radioactivity after tritiation due to the possible introduction of two tritium atoms. The final tritiation of the brominated precursor was performed by catalytic hydrogenation (custom-labeling), which led to the displacement of the bromine by tritium atoms (see Fig. 3).

[^3^H]PSB-OR-2020 was obtained in high purity (97.7%). It showed a specific activity of 45 Ci/mmol (1.67 TBq/mmol) indicating a good tritiation efficiency (introduction of ca. 1.5 tritium atoms per molecule, for analytical details see Figs. S1 and S2 of Supporting Information).

### Development of a radioligand binding assay

**Fig. 4:**
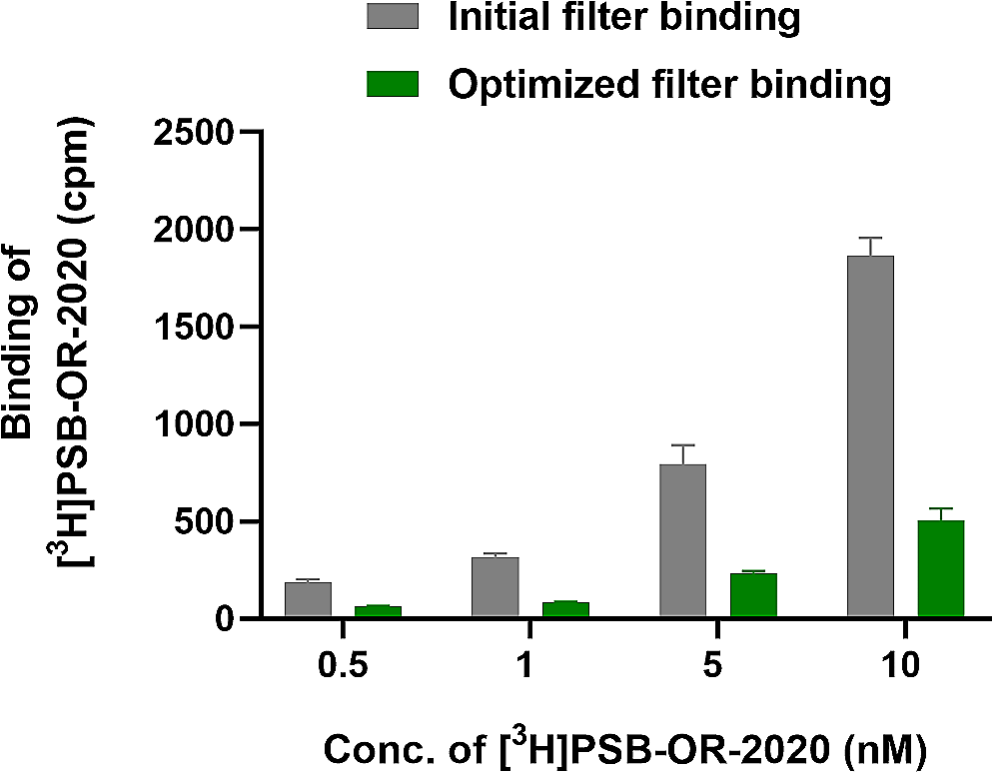
Optimization of filter binding. Gray bars: initial GF/B filter binding of [^3^H]PSB-OR-2020 (assay buffer: ice-cold 50 mM Tris-HCl, pH 7.4; assay volume: 1 mL; washing buffer: ice-cold 50 mM Tris-HCl, pH 7.4 containing 0.1% BSA; incubation on ice for 30 min; filter was dried at room temperature for 30 min). Green bars: optimized assay conditions showing decreased non-specific radioligand binding to GF/C filters (assay buffer: ice-cold 50 mM Tris-HCl, pH 7.4 containing 0.1% BSA; assay volume: 500 µL; washing buffer: ice-cold 50 mM Tris-HCl, pH 7.4; incubation on ice for 30 min; filter was dried at 70 °C for 60–90 min)

As a next step, the new radioligand was utilized to establish a P2X4 receptor radioligand binding assay. The initially observed binding of [^3^H]PSB-OR-2020 to GF/B glass fiber filters was relatively high (up to 2000 cpm at a radioligand concentration of 10 nM) using ice-cold Tris-HCl buffer (50 mM, pH 7.4) in a final volume of 1 mL at 4 °C, and ice-cold Tris-HCl buffer (50 mM, pH 7.4) containing 0.1% bovine serum albumin (BSA) as washing buffer. The GF/B filters were dried for 30 min at room temperature before transferring them into scintillation vials. To reduce filter binding, several experimental parameters were varied, including buffer, additives, filter types, and filter pretreatment. Reduced filter binding (to ca. 500 cpm at a radioligand concentration of 10 nM) was achieved by using an assay buffer composed of 50 mM Tris-HCl, pH 7.4, containing 0.1% BSA, incubation at 4 °C, a washing buffer consisting of ice-cold 50 mM Tris-HCl, pH 7.4, and filtration through GF/C filters, which were dried at 70 °C for 60–90 min after harvesting (see Fig. 4). GF/C glass fiber filters are thinner and have a larger pore size of 1.2 μm, in comparison to GF/B filters (1.0 μm).

As a next step, experiments with membrane preparations derived from recombinant, P2X4 receptor-expressing 1321N1 astrocytoma cells were performed. In these cells, the human P2X4 receptor was stably expressed using a retroviral expression system [49]. Non-specific binding was determined in the presence of unlabeled PSB-OR-2020 (either 500 nM or 10 µM). Preliminary experiments using 3 nM or 7 nM of [^3^H]PSB-OR-2020, respectively, indicated specific binding of [^3^H]PSB-OR-2020 to the P2X4 receptor-expressing cell membrane preparations, but non-specific binding was still high (> 50% of total binding, data not shown).

To decrease non-specific radioligand binding to the cell membrane preparations, various compositions of assay and washing buffer were tested. The washing buffer was supplemented with magnesium chloride, copper chloride, ethylenediamine tetraacetic acid (EDTA), ethylene glycol tetraacetic acid (EGTA), acetic acid or sodium phosphate. Since the radioligand is well soluble in ethanol, 5% of ethanol were added to the washing buffer (ice-cold 50 mM Tris-HCl, pH 7.4) to remove unbound radioligand. Preincubation of GF/C filters in polyethylenimine solutions of different concentrations, in trimethylchlorosilane solution, or in washing buffer containing 5% of ethanol, was investigated. However, none of these modifications led to a sufficient reduction in non-specific radioligand binding, and the percentage of specific binding of [^3^H]PSB-OR-2020 was still too low. The radioligand’s non-specific binding appeared to be mainly attributed to binding non-specifically to the membrane preparation, while filter binding was moderate after it had been optimized. The low percentage of specific radioligand binding may additionally be explained by a rather low expression level of the P2X4 receptor.

Thus, new cell lines were created with the aim to obtain higher receptor expression. Membrane preparations of the resulting cell lines were used for radioligand binding studies to select the most highly expressing, and thus best suitable one. In addition to the previously described 1321N1 astrocytoma cell line, two further P2X4 receptor-expressing cell lines were studied: (i) HEK293 cells transiently expressing the human P2X4 receptor (plasmid: pQCXIN), (ii) HEK293 cells transiently expressing the human P2X4 receptor (plasmid: pcDNA3.1(-)). HEK293 cells transiently expressing the human P2X4 receptor (plasmid: pQCXIN) showed a higher expression level and resulted in a consistent, reproducible, and sufficiently low percentage of non-specific binding.

The optimal conditions for the binding assays were as follows: a final volume of 500 µL of Tris-HCl buffer (50 mM), pH 7.4, containing 0.1% BSA, 10 nM radioligand, 50–150 µg of protein (membrane preparation of HEK293 cells recombinantly expressing P2X4), incubation for 60 min at 4 °C, filtration through GF/C filters, and washing with ice-cold Tris-HCl buffer (50 mM), pH 7.4, containing 5% ethanol. Non-specific binding was determined in the presence of unlabeled PSB-OR-2020 (10 µM).

**Fig. 5:**
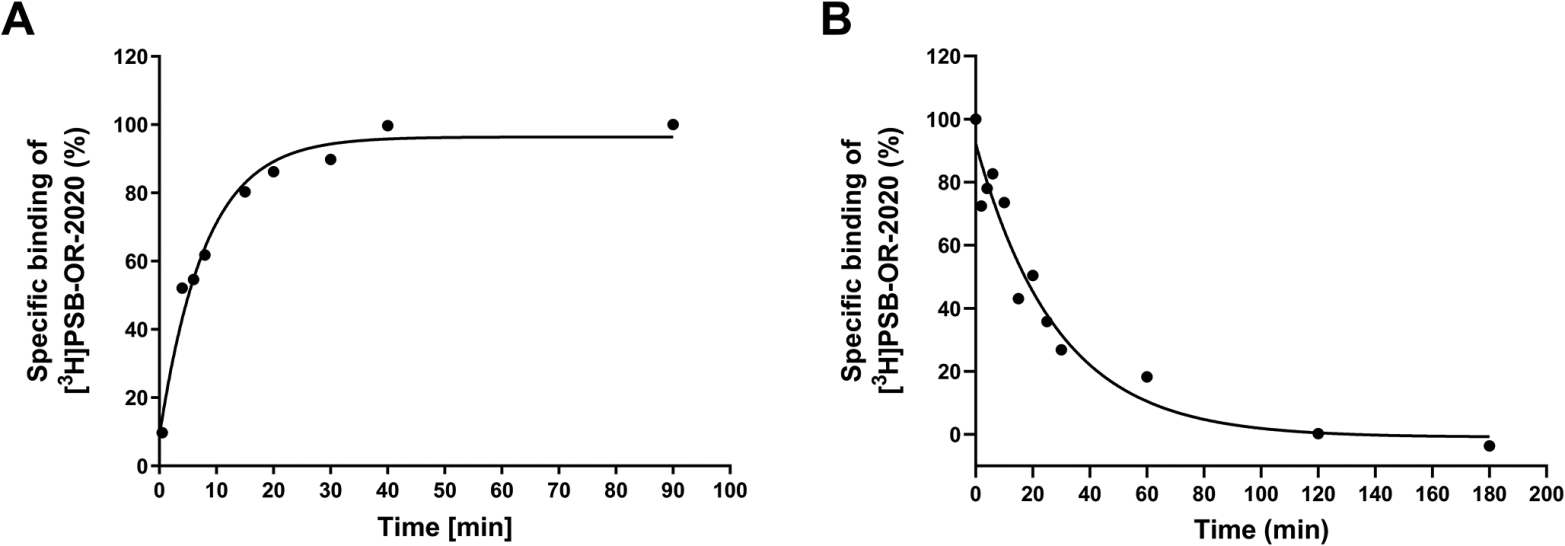
Representative curves for association (**A**) and dissociation (**B**) of [^3^H]PSB-OR-2020 (10 nM) to HEK293 cell membrane preparations recombinantly expressing the human P2X4 receptor, performed at 4 °C

To characterize the binding kinetics of the radioligand, we performed association and dissociation experiments at 4 °C (see Fig. 5A and B). [^3^H]PSB-OR-2020 associated with a half-life of 8.44 ± 2.59 min, while the dissociation half-life was 20.4 ± 2.9 min. Three independent association and dissociation experiments were performed to determine the kinetic K_D_ value (k_off_/k_on_) for [^3^H]PSB-OR-2020, which was calculated to be 7.06 ± 3.23 nM.

**Fig. 6:**
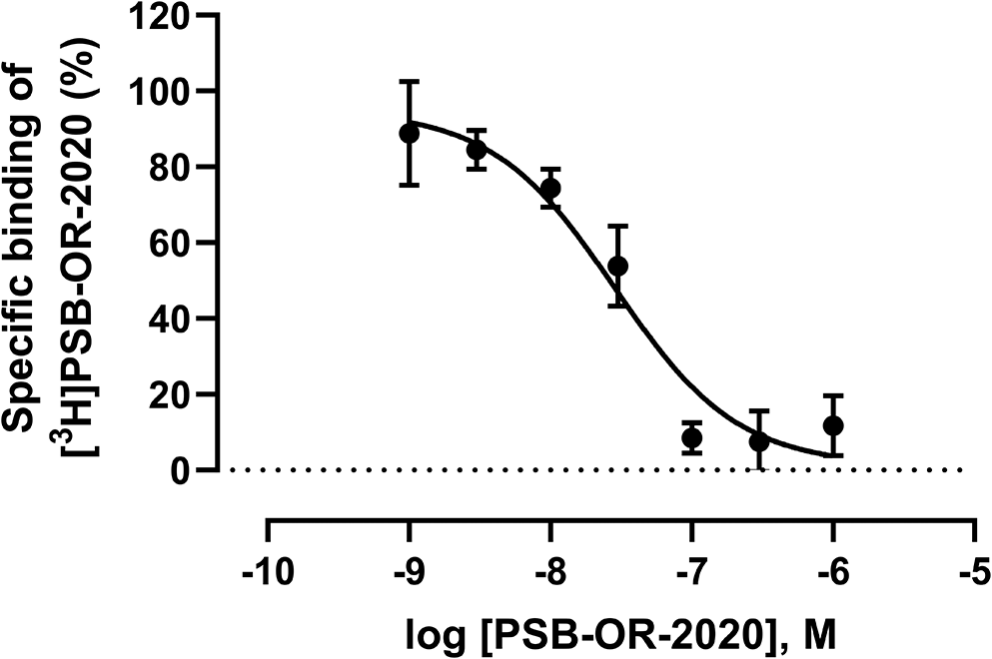
Competition binding of different concentrations of PSB-OR-2020 vs. [^3^H]PSB-OR-2020 (10 nM) using membrane preparations of HEK293 cells recombinantly expressing the human P2X4 receptor. Data points represent means ± SEM of three independent experiments performed in duplicates. K_D_ value of PSB-OR-2020: 20.4 ± 7.5 nM; B_max_ value: 71.7 fmol/mg of protein

We subsequently performed homologous competition binding studies, and determined a concentration-inhibition curve for the unlabeled ligand PSB-OR-2020 (see Fig. 6). A K_D_ value of 20.4 ± 7.5 nM was calculated from three independent experiments performed in duplicates. The B_max_ value was 71.7 fmol/mg of protein, and thus still not very high. The K_D_ value correlated well with the kinetic K_D_ value (7.06 ± 3.23 nM) and also with the IC_50_ value of the unlabeled PSB-OR-2020 determined in calcium influx assays (6.32 ± 1.52 nM) versus the EC_80_ concentration of the agonist ATP (300 nM).

**Fig. 7:**
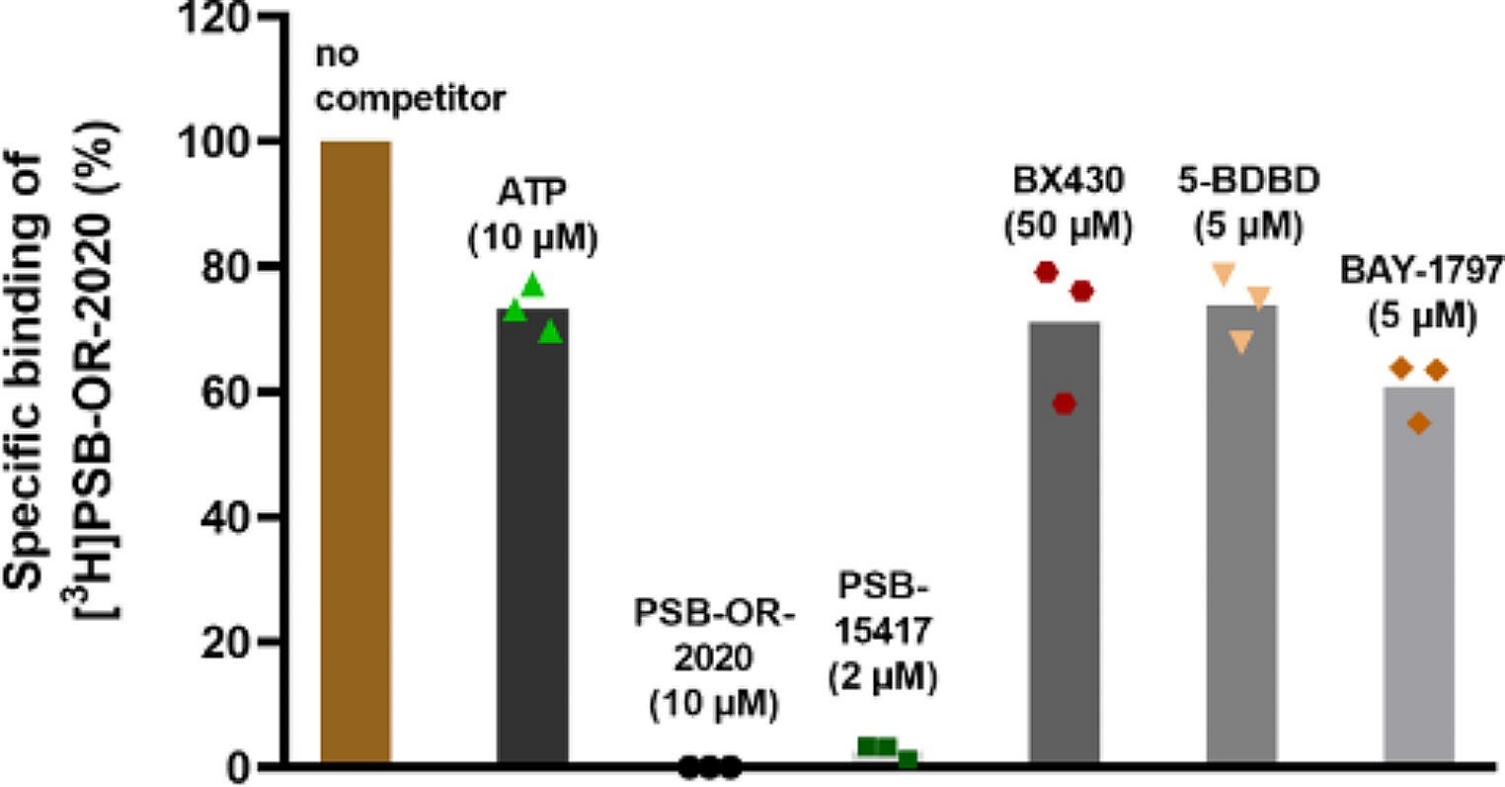
Specific [^3^H]PSB-OR-2020 binding to the human P2X4 receptor in the absence and presence of ATP and various P2X4 receptor antagonists utilizing membrane preparations of HEK293 cells transiently transfected with the human P2X4 receptor. Data represent means of three independent experiments performed in duplicates and triplicates. The final concentrations of the compounds are shown in brackets

As a next step, the effects of structurally diverse modulators of the human P2X4 receptor were studied (Fig. 7). Based on the IC_50_ values determined in calcium influx assays, and considering the compounds’ solubilities, high concentrations were employed that were expected to result in a complete blockade of the receptor. PSB-15417 completely displaced the radioligand from its binding site, suggesting that the radioligand and PSB-15417 (both being structurally related compounds) bind to the same allosteric site of the human P2X4 receptor. In contrast, a high concentration of ATP only slightly displaced the radioligand from its binding site, providing evidence for an allosteric binding mode of [^3^H]PSB-OR-2020 (Fig. 7). The structurally diverse P2X4 receptor antagonists BX430, 5-BDBD, and BAY-1797 (for structures see Fig. 1) also only partially displaced the radioligand from its binding site by less than 40%, even though high concentrations were employed. Thus, we assume that 5-BDBD, BX430 and BAY-1797 bind to a site or sites different from that of [^3^H]PSB-OR-2020.

## Discussion

Purinergic signaling is involved in many physiological processes [52]. The balance between extracellular concentrations of pro-inflammatory ATP and anti-inflammatory adenosine is crucial for homeostasis [53, 54]. Under inflammatory conditions, extracellular ATP concentrations may rise leading to the activation of P2X receptors [55]. These receptors are currently in the focus of interest as novel drug targets. The first P2X3 receptor antagonist, gefapixant, has recently been approved for the treatment of chronic cough, characterized by lung inflammation [56, 57]. P2X4 receptor antagonists are potential drug targets for inflammatory and neurodegenerative diseases including neuropathic pain [58], inflammatory pain [58], Parkinson’s disease [22], Alzheimer’s disease [21], multiple sclerosis [59], epilepsy [24], and cancer [28–30]. To study P2X4 receptor pharmacology, suitable tool compounds are required. Up to now, no radio- or fluorescence-labeled antagonist suitable for P2X4 receptor binding studies has been developed. This might be attributed to the fact that most of the P2X4 receptor antagonists described so far are only moderately potent and/or display low water-solubility resulting in high non-specific binding to cell membranes. In the present study, we describe the development of the tritium-labeled P2X4 receptor antagonist [^3^H]PSB-OR-2020 belonging to a novel chemical class of allosteric P2X4 receptor antagonists, and present its preliminary characterization. Major hurdles on the way to develop a binding assay were the compound’s relatively high glass fiber filter binding, and its non-specific binding to cell membranes. Moreover, it was hampered by the low expression of the P2X4 receptor in the available cell lines. Nevertheless, we managed to perform kinetic studies to determine the radioligand’s kinetic K_D_ value of 7.06 nM, and competition binding assays resulting in a K_D_ value of 20.4 nM. These data confirmed the high affinity of [^3^H]PSB-OR-2020, which is well in agreement with its IC_50_ value obtained in calcium influx assays.

In future studies, more highly expressing cell lines, or isolated P2X4 receptor protein reconstituted into nanodiscs or amphipols, could be advantageous to further increase specific binding and to reduce non-specific binding.

Our radioligand represents an allosteric P2X4 receptor antagonist with a novel chemical structure. ATP does not compete with its binding to the receptor. Since the radioligand could also not or only partially be displaced by some previously published P2X4 receptor antagonists, including BX430, 5-BDBD, and BAY-1797, we assume that those antagonists bind to different allosteric binding sites.

Different allosteric binding sites, besides the orthosteric site, have been described for P2X receptor antagonists, as revealed by X-ray crystallography, cryo-EM, and mutagenesis studies [5, 36, 60, 61, 62]. Recently, the allosteric binding site of BX430 and BAY-1797 on the zebrafish P2X4 receptor was determined by cryo-EM to be located in the extracellular domain of the receptor, at the inter-subunit interface of the receptor trimer [5]. Our competition binding assays showed that the binding site of the new radioligand [^3^H]PSB-OR-2020 at the human P2X4 receptor, to which its structurally related antagonist PSB-15417 binds, likely differs from the allosteric binding site of the structurally different P2X4 receptor antagonists BX430 and BAY-1797, and thus appears to occupy a novel allosteric site.

## Conclusion

In conclusion, we have successfully designed and synthesized a P2X4 receptor radioligand which allowed for the first time to establish a binding assay for this therapeutically relevant receptor. [^3^H]PSB-OR-2020 may become a useful tool in P2X4 receptor pharmacology and drug development.

## Methods

### Chemicals

Chemicals were obtained from Roth (Karlsruhe, Germany), AppliChem (Darmstadt, Germany) or Thermo Fisher Scientific (Darmstadt, Germany). The radioligand [^3^H]PSB-OR-2020 (45 Ci/mmol; 1.67 TBq/mmol) was produced by custom-labeling (Pharmaron UK Ltd., Cardiff, UK), see Supporting Information.

### Retroviral transfection

The 1321N1 astrocytoma cell line stably expressing the human P2X4 receptor was generated using GP + envAM-12 fibroblast-type mouse packaging cells to produce a recombinant helper virus as described before [63]. To produce recombinant helper viruses, 6.25 µg of the plasmid DNA (human P2X4 receptor DNA in pQCXIN) and 3.75 µg of vesicular stomatitis virus G (VSV-G) protein DNA (in pcDNA3.1) were combined. Subsequently, packaging cells were transfected using lipofectamine 2000 (Invitrogen, Carlsbad, CA, USA) as a transfection reagent. After harvesting the virus particles, the target cells (non-transfected 1321N1 astrocytoma cells) were infected. 48–72 h after infection, the selection was started using the selection marker G418 (800 µg/mL). Cells were cultured in Dulbecco’s Modified Eagle medium (DMEM) (Thermo Fisher Scientific, Darmstadt, Germany), 10% fetal calf serum (FCS) (PAN Biotech, Aidenbach, Germany), a mixture of penicillin/streptomycin (100 U/mL penicillin, 100 µg/mL streptomycin) (PAN Biotech, Aidenbach, Germany), and the selection antibiotic geneticin G418 (800 µg/mL). Cells were grown at 37 °C and 10% CO_2_.

### Transient transfection

HEK293 cells were transiently transfected with the human P2X4 DNA in pQCXIN or pcDNA3.1(-). Transfection was conducted with 10 µg of the plasmid DNA and a mixture of Opti-MEM™ medium (Thermo Fisher Scientific, Darmstadt, Germany) and lipofectamine 2000 (Invitrogen, Carlsbad, CA, USA) that served as the transfection reagent. The selection was started after 24–72 h using DMEM, 10% FCS, a mixture of penicillin/streptomycin (100 U/mL penicillin, 100 µg/mL streptomycin) and G418 (800 µg/mL). Cells were grown at 37 °C and 5% CO_2_.

### Membrane preparation

Cells expressing the human P2X4 receptor were grown to 70–90% confluence in cell culture dishes at 37 °C and 10% CO_2_. After removing the medium, the cells were washed with phosphate buffer (phosphate-buffered saline (PBS)), and frozen at -20 °C. The next day, the cells were scraped off the dishes after adding 1 mL of cold hypotonic buffer (50 mM Tris-HCl, 1 mM EDTA, pH 7.4) per dish. All steps were carried out on ice. After homogenizing the cell suspension using an UltraTurrax® (IKA Labortechnik, Staufen, Germany), the homogenate was centrifuged for 10 min at 1,000 *g* and 4 °C. The supernatant was centrifuged again for 60 min at 48,000 *g* and 4 °C. The remaining pellet was resuspended in 50 mM Tris-HCl buffer, pH 7.4, and stored in aliquots at -80 °C.

### Protein concentration determination with the Bradford assay [64]

To estimate the protein concentration of the membrane preparations, a Bradford assay was performed. The Bradford stock solution contained 0.1 g of Coomassie-Brillant Blue G dissolved in 50 mL ethanol (v/v) with 100 mL of 85% phosphoric acid, diluted to 1000 mL with water. A BSA calibration curve (100 µg/mL − 500 µg/mL) was prepared in 50 mM Tris-HCl buffer, pH 7.4. The membrane preparations were diluted 1:10 and 1:20 with 50 mM Tris-HCl buffer, pH 7.4. Then, 1000 µL of the Bradford stock solution and 50 µL of the sample were mixed in a cuvette. The samples were measured at 595 nm and the final protein concentration was determined based on a BSA calibration curve.

### Radioligand binding studies

Kinetic and competition binding studies using membrane preparations from HEK293 cells recombinantly expressing the human P2X4 receptor were performed in 50 mM Tris-HCl, pH 7.4, and 0.1% BSA at 4 °C, in a final assay volume of 500 µL. The assay vials contained either 5 µL of the test compound (dissolved in dimethyl sulfoxide (DMSO)) or 5 µL of DMSO, 50 µL of [^3^H]PSB-OR-2020 in assay buffer (10 nM), and 50 µL of protein in 50 mM Tris-HCl buffer, pH 7.4. Non-specific and total binding of [^3^H]PSB-OR-2020 were determined using 10 µM of unlabeled PSB-OR-2020 dissolved in DMSO, and 5 µL of DMSO, respectively. To adjust a suitable amount of protein, the specific binding of the radioligand was measured for each newly generated membrane preparation. After adding the cell membrane suspension (50–150 µg of protein), the incubation was started and lasted for 60 min at 4 °C with gentle shaking. For association experiments, 50 µL of the cell membrane suspension were added at specific time points over a 90 min period. For dissociation experiments, the radioligand was first incubated with the cell membrane suspension for 1 h. Then, 5 µL of unlabeled PSB-OR-2020 (10 µM) were added at specific time points (ranging from 180 min to 0 min) to initiate dissociation. The incubation was terminated by rapid vacuum filtration using a Brandel 24-well harvester (Brandel, Gaithersburg, MD, USA), and washing with 3 × 3 mL of ice-cold washing buffer (50 mM Tris-HCl buffer, pH 7.4, containing 5% ethanol) through Whatman GF/C glass fiber filters (GE Healthcare Life Sciences Whatman™, USA). The filters were preincubated in ice-cold washing buffer for 30 min. After filtration, the filters were punched out and soaked in 2.5 mL of scintillation cocktail (ProSafe FC+, Meridian Biotechnologies Ltd., Surrey, UK) for at least 6 h before being counted using a liquid scintillation counter (Tri-Carb® 2900 TR, Perkin-Elmer, Rodgau, Germany, 53% efficiency).

### Calcium influx assay

To determine the effects of the compound on P2X4, P2X2, and P2X7 receptors, the agonist-mediated increases in cytosolic Ca^2+^ concentrations were measured using the fluorescent Ca^2+^ chelating dye Fluo-4 acetoxymethyl ester (AM) (Thermo Fisher Scientific). On the first day, 1321N1 astrocytoma cells (45.000 cells/well) stably expressing the respective P2X receptor were seeded into a 96-well plate (No. 3340, Corning, Kennebunk, Maine, USA), and incubated at 37 °C and 10% of CO_2_. The next day, the cells were incubated with Fluo-4 AM (3 µM) in Hanks’ balanced salt solution and 1% of Pluronic® F127 (Sigma Aldrich, St. Louis, MO, USA) for 1 h at room temperature with slight shaking. For human P2X7 receptors, a different assay buffer was used (150 mM Na-glutamate, 5 mM KCl, 0.5 mM CaCl_2_, 0.1 mM MgCl_2_, 10 mM D-glucose, 25 mM HEPES, pH 7.4). Calcium 5 (Molecular Devices, San Jose, CA, USA) was used as a fluorescent dye to investigate recombinantly expressed human P2X1 and P2X3 receptors. The cells were loaded with Calcium 5 dye in HBSS buffer, and incubated for 1 h at 37 °C and 10% CO_2_ for human P2X1, and 5% CO_2_ for human P2X3 receptors.

Following the incubation, the dye solution was carefully removed, and the antagonist (dissolved in DMSO) and HBSS buffer were added to the cells. The final DMSO concentration was 1% for human P2X4, P2X2, and P2X7 receptors, and 0.5% for human P2X1 and P2X3 receptors. To activate the receptors, transparent 96-well reagent plates (Boettger, Bodenmais, Germany) containing the agonists dissolved in HBSS buffer (10-fold concentration of previously determined EC_80_) were prepared. The plates were measured after 30 min of preincubation with antagonist with the imaging plate reader NOVOstar (BMG Labtech GmbH, Offenburg, Germany) at 520 nm for 30 s at 0.4 s intervals.

### Data analysis

Data were analyzed using Microsoft Excel and PRISM Version 8.0 (GraphPad Prism 8, San Diego, CA).

## Electronic supplementary material

Below is the link to the electronic supplementary material.

Supplementary Material 1

## Data Availability

Data is provided within the manuscript or supplementary information files. The authors filed a patent on the new class of P2X4 receptor antagonists including the radioligand described in this study.

## Abbreviations

ALS: Amyotrophic lateral sclerosis
AM: Acetoxymethyl ester
ATP: Adenosine 5’-triphosphate
BDNF: Brain-derived neurotrophic factor
BSA: Bovine serum albumin
Bz-ATP: 2’(3’)-O-(4-benzoylbenzoyl) adenosine-5’-triphosphate
CNS: Central nervous system
cpm: Counts per minute
Cryo-EM: Cryogenic electron microscopy
DMEM: Dulbecco’s Modified Eagle Medium
DMSO: Dimethyl sulfoxide
EDTA: Ethylenediamine tetraacetic acid
EGTA: Ethylene glycol tetraacetic acid
FCS: Fetal calf serum
G418: Geneticin
GF/C: Glass fiber filters
HBSS: Hanks’ balanced salt solution
HEK293: Human embryonic kidney type 293
HEPES: 4-(2-Hydroxyethyl)-1-piperazineethanesulfonic acid
IC_50_: Half-maximal inhibitory concentration
K_D_: Dissociation constant
PET: Positron emission tomography
PBS: Phosphate-buffered saline
SEM: Standard error of the mean
SPECT: Single photon emission computer tomography
TM: Transmembrane domain
Tris-HCl: Tris(hydroxymethyl)aminomethane hydrochloride
VSV-G: Vesicular stomatitis virus G

## References

[1] Illes P, Müller CE, Jacobson KA, Grutter T, Nicke A, Fountain SJ, Kennedy C, Schmalzing G, Jarvis MF, Stojilkovic SS, King BF, Di Virgilio F (2021) Update of P2X receptor properties and their pharmacology: IUPHAR Review 30. Br J Pharmacol 178:489–514. 10.1111/bph.15299 33125712 10.1111/bph.15299 PMC8199792

[2] Kawate T, Michel JC, Birdsong WT, Gouaux E (2009) Crystal structure of the ATP-gated P2X4 ion channel in the closed state. Nature 460:592–598. 10.1038/nature08198 19641588 10.1038/nature08198 PMC2720809

[3] Hattori M, Gouaux E (2012) Molecular mechanism of ATP binding and ion channel activation in P2X receptors. Nature 485:207–212. 10.1038/nature11010 22535247 10.1038/nature11010 PMC3391165

[4] Saul A, Hausmann R, Kless A, Nicke A (2013) Heteromeric assembly of P2X subunits. Front Cell Neurosci 7:250. 10.3389/fncel.2013.00250 24391538 10.3389/fncel.2013.00250 PMC3866589

[5] Shen C, Zhang Y, Cui W, Zhao Y, Sheng D, Teng X, Shao M, Ichikawa M, Wang J, Hattori M (2023) Structural insights into the allosteric inhibition of P2X4 receptors. Nat Commun 14:6437. 10.1038/s41467-023-42164-y 37833294 10.1038/s41467-023-42164-y PMC10575874

[6] North RA (2016) P2X receptors. Phil Trans R Soc 371:20150427. 10.1098/rstb.2015.0427 10.1098/rstb.2015.0427 PMC4938027 27377721

[7] Schneider M, Prudic K, Pippel A, Klapperstück M, Braam U, Müller CE, Schmalzing G, Markwardt F (2017) Interaction of purinergic P2X4 and P2X7 receptor subunits. Front Pharmacol 8:860. 10.3389/fphar.2017.00860 29213241 10.3389/fphar.2017.00860 PMC5702805

[8] Sophocleous RA, Ooi L, Sluyter R (2022) The P2X4 receptor: cellular and molecular characteristics of a promising neuroinflammatory target. Int J Mol Sci 23:5739. 10.3390/ijms23105739 35628550 10.3390/ijms23105739 PMC9147237

[9] Stokes L, Layhadi JA, Bibic L, Dhuna K, Fountain SJ (2017) P2X4 receptor function in the nervous system and current breakthroughs in pharmacology. Front Pharmacol 8:291. 10.3389/fphar.2017.00291 28588493 10.3389/fphar.2017.00291 PMC5441391

[10] Tsuda M, Masuda T, Tozaki-Saitoh H, Inoue K (2013) P2X4 receptors and neuropathic pain. Front Cell Neurosci 7:191. 10.3389/fncel.2013.00191 24191146 10.3389/fncel.2013.00191 PMC3808787

[11] Ulmann L, Hatcher JP, Hughes JP, Chaumont S, Green PJ, Conquet F, Buell GN, Reeve AJ, Chessell IP, Rassendren F (2008) Up-regulation of P2X4 receptors in spinal microglia after peripheral nerve injury mediates BDNF release and neuropathic pain. J Neurosci 28:11263–11268. 10.1523/JNEUROSCI.2308-08.2008 18971468 10.1523/JNEUROSCI.2308-08.2008 PMC6671487

[12] Inoue K (2006) The function of microglia through purinergic receptors: neuropathic pain and cytokine release. Pharmacol Ther 109:210–226. 10.1016/j.pharmthera.2005.07.001 16169595 10.1016/j.pharmthera.2005.07.001

[13] Inoue K (2019) Role of the P2X4 receptor in neuropathic pain. Curr Opin Pharmacol 47:33–39. 10.1016/j.coph.2019.02.001 30878800 10.1016/j.coph.2019.02.001

[14] Teixeira JM, dos Santos GG, Neves AF, Athie MCP, Bonet IJM, Nishijima CM, Farias FH, Figueiredo JG, Hernandez-Olmos V, Alshaibani S, Tambeli CH, Müller CE, Parada CA (2019) Diabetes-induced neuropathic mechanical hyperalgesia depends on P2X4 receptor activation in dorsal root ganglia. Neurosci 398:158–170. 10.1016/j.neuroscience.2018.12.003 10.1016/j.neuroscience.2018.12.003 30537520

[15] Kohno K, Tsuda M (2021) Role of microglia and P2X4 receptors in chronic pain. Pain Rep 6:e864. 10.1097/PR9.0000000000000864 33981920 10.1097/PR9.0000000000000864 PMC8108579

[16] Tsuda M, Shigemoto-Mogami Y, Koizumi S, Mizokoshi A, Kohsaka S, Salter MW, Inoue K (2003) P2X4 receptors induced in spinal microglia gate tactile allodynia after nerve injury. Nature 424:778–783. 10.1038/nature01786 12917686 10.1038/nature01786

[17] Franceschini A, Adinolfi E (2014) P2X receptors: new players in cancer pain. World J Biol Chem 5:429–436. 10.4331/wjbc.v5.i4.429 25426266 10.4331/wjbc.v5.i4.429 PMC4243147

[18] Ulmann L, Hirbec H, Rassendren F (2010) P2X4 receptors mediate PGE2 release by tissue-resident macrophages and initiate inflammatory pain. EMBO J 29:2290–2300. 10.1038/emboj.2010.126 20562826 10.1038/emboj.2010.126 PMC2910276

[19] Li F, Guo N, Ma Y, Ning B, Wang Y, Kou L (2014) Inhibition of P2X4 suppresses joint inflammation and damage in collagen-induced arthritis. Inflammation 37:146–153. 10.1007/s10753-013-9723-y 24062058 10.1007/s10753-013-9723-y

[20] Chen L, Liu Y, Yue K, Ru Q, Xiong Q, Ma B, Tian X, Li C (2016) Differential expression of ATP-gated P2X receptors in DRG between chronic neuropathic pain and visceralgia rat models. Purinergic Signal 12:79–87. 10.1007/s11302-015-9481-4 26531254 10.1007/s11302-015-9481-4 PMC4749526

[21] Varma R, Chai Y, Troncoso J, Gu J, Xing H, Stojilkovic SS, Mattson MP, Haughey NJ (2009) Amyloid-beta induces a caspase-mediated cleavage of P2X4 to promote purinotoxicity. Neuromolecular Med 11:63–75. 10.1007/s12017-009-8073-2 19562525 10.1007/s12017-009-8073-2 PMC2735730

[22] Zhang X, Wang J, Gao J-Z, Zhang X-N, Dou K-X, Shi W-D, Xie A-M (2021) P2X4 receptor participates in autophagy regulation in Parkinson’s disease. Neural Regen Res 16:2505–2511. 10.4103/1673-5374.313053 33907041 10.4103/1673-5374.313053 PMC8374561

[23] Bertin E, Martinez A, Fayoux A, Carvalho K, Carracedo S, Fernagut P-O, Koch-Nolte F, Blum D, Bertrand SS, Boué-Grabot E (2022) Increased surface P2X4 receptors by mutant SOD1 proteins contribute to ALS pathogenesis in SOD1-G93A mice. Cell Mol Life Sci 79:431. 10.1007/s00018-022-04461-5 35852606 10.1007/s00018-022-04461-5 PMC9296432

[24] Ulmann L, Levavasseur F, Avignone E, Peyroutou R, Hirbec H, Audinat E, Rassendren F (2013) Involvement of P2X4 receptors in hippocampal microglial activation after status epilepticus. Glia 61:1306–1319. 10.1002/glia.22516 23828736 10.1002/glia.22516

[25] Zabala A, Vazquez-Villoldo N, Rissiek B, Gejo J, Martin A, Palomino A, Perez-Samartín A, Pulagam KR, Lukowiak M, Capetillo-Zarate E, Llop J, Magnus T, Koch-Nolte F, Rassendren F, Matute C, Domercq M (2018) P2X4 receptor controls microglia activation and favors remyelination in autoimmune encephalitis. EMBO Mol Med 10:e8743. 10.15252/emmm.201708743 29973381 10.15252/emmm.201708743 PMC6079537

[26] Verma R, Cronin CG, Hudobenko J, Venna VR, McCullough LD, Liang BT (2017) Deletion of the P2X4 receptor is neuroprotective acutely, but induces a depressive phenotype during recovery from ischemic stroke. Brain Behav Immun 66:302–312. 10.1016/j.bbi.2017.07.155 28751018 10.1016/j.bbi.2017.07.155 PMC5650951

[27] Srivastava P, Cronin CG, Scranton VL, Jacobson KA, Liang BT, Verma R (2020) Neuroprotective and neuro-rehabilitative effects of acute purinergic receptor P2X4 (P2X4R) blockade after ischemic stroke. Exp Neurol 329:113308. 10.1016/j.expneurol.2020.113308 32289314 10.1016/j.expneurol.2020.113308 PMC7242087

[28] Chadet S, Allard J, Brisson L, Lopez-Charcas O, Lemoine R, Heraud A, Lerondel S, Guibon R, Fromont G, Le Pape A, Angoulvant D, Jiang L-H, Murrell-Lagnado R, Roger S (2022) P2X4 receptor promotes mammary cancer progression by sustaining autophagy and associated mesenchymal transition. Oncogene 41:2920–2931. 10.1038/s41388-022-02297-8 35411034 10.1038/s41388-022-02297-8

[29] Maynard JP, Lu J, Vidal I, Hicks J, Mummert L, Ali T, Kempski R, Carter AM, Sosa RY, Peiffer LB, Joshu CE, Lotan TL, de Marzo AM, Sfanos KS (2022) P2X4 purinergic receptors offer a therapeutic target for aggressive prostate cancer. J Pathol 256:149–163. 10.1002/path.5815 34652816 10.1002/path.5815 PMC8738159

[30] Reyna-Jeldes M, La Fuente-Ortega E, Cerda D, Velázquez-Miranda E, Pinto K, Vázquez-Cuevas FG, Coddou C (2021) Purinergic P2Y2 and P2X4 receptors are involved in the epithelial-mesenchymal transition and metastatic potential of gastric cancer derived cell lines. Pharmaceutics 13:1234. 10.3390/pharmaceutics13081234 34452195 10.3390/pharmaceutics13081234 PMC8398939

[31] Schmitt M, Ceteci F, Gupta J, Pesic M, Böttger TW, Nicolas AM, Kennel KB, Engel E, Schewe M, Callak Kirisözü A, Petrocelli V, Dabiri Y, Varga J, Ramakrishnan M, Karimova M, Ablasser A, Sato T, Arkan MC, de Sauvage FJ, Greten FR (2022) Colon tumour cell death causes mTOR dependence by paracrine P2X4 stimulation. Nature 612:347–353. 10.1038/s41586-022-05426-1 36385525 10.1038/s41586-022-05426-1 PMC7613947

[32] Rupert C, Dell’ Aversana C, Mosca L, Montanaro V, Arcaniolo D, de Sio M, Bilancio A, Altucci L, Palinski W, Pili R, de Nigris F (2023) Therapeutic targeting of P2X4 receptor and mitochondrial metabolism in clear cell renal carcinoma models. J Exp Clin Cancer Res 42:134. 10.1186/s13046-023-02713-1 37231503 10.1186/s13046-023-02713-1 PMC10214673

[33] Brock VJ, Wolf IMA, Er-Lukowiak M, Lory N, Stähler T, Woelk L-M, Mittrücker H-W, Müller CE, Koch-Nolte F, Rissiek B, Werner R, Guse AH, Diercks B-P (2022) P2X4 and P2X7 are essential players in basal T cell activity and Ca^2+^ signaling milliseconds after T cell activation. Sci Adv 8:eabl9770. 10.1126/sciadv.abl9770 35119925 10.1126/sciadv.abl9770 PMC8816335

[34] Coddou C, Stojilkovic SS, Huidobro-Toro JP (2011) Allosteric modulation of ATP-gated P2X receptor channels. Rev Neurosci 22:335–354. 10.1515/rns.2011.014 21639805 10.1515/RNS.2011.014 PMC3647606

[35] Evans RJ (2009) Orthosteric and allosteric binding sites of P2X receptors. Eur Biophys J 38:319–327. 10.1007/s00249-008-0275-2 18247022 10.1007/s00249-008-0275-2

[36] Müller CE, Namasivayam V (2021) Recommended tool compounds and drugs for blocking P2X and P2Y receptors. Purinergic Signal 17:633–648. 10.1007/s11302-021-09813-7 34476721 10.1007/s11302-021-09813-7 PMC8677864

[37] Matsumura Y, Yamashita T, Sasaki A, Nakata E, Kohno K, Masuda T, Tozaki-Saitoh H, Imai T, Kuraishi Y, Tsuda M, Inoue K (2016) A novel P2X4 receptor-selective antagonist produces anti-allodynic effect in a mouse model of herpetic pain. Sci Rep 6:32461. 10.1038/srep32461 27576299 10.1038/srep32461 PMC5006034

[38] Toti KS, Verma R, McGonnigle MJ, Gamiotea Turro D, Wen Z, Lewicki SA, Liang BT, Jacobson KA (2022) Structure-activity relationship and neuroprotective activity of 1,5-Dihydro-2*H*-naphtho[1,2-*b*][1,4]-diazepine-2,4(3*H*)-diones as P2X4 receptor antagonists. J Med Chem 65:13967–13987. 10.1021/acs.jmedchem.2c01197 36150180 10.1021/acs.jmedchem.2c01197 PMC9653265

[39] Hernandez-Olmos V, Abdelrahman A, El-Tayeb A, Freudendahl D, Weinhausen S, Müller CE (2012) *N*-substituted phenoxazine and acridone derivatives: structure-activity relationships of potent P2X4 receptor antagonists. J Med Chem 55:9576–9588. 10.1021/jm300845v 23075067 10.1021/jm300845v

[40] Werner S, Mesch S, Hillig RC, Ter Laak A, Klint J, Neagoe I, Laux-Biehlmann A, Dahllöf H, Bräuer N, Puetter V, Nubbemeyer R, Schulz S, Bairlein M, Zollner TM, Steinmeyer A (2019) Discovery and characterization of the potent and selective P2X4 inhibitor N-4-(3-Chlorophenoxy)-3-sulfamoylphenyl-2-phenylacetamide (BAY-1797) and structure-guided amelioration of its CYP3A4 induction profile. J Med Chem 62:11194–11217. 10.1021/acs.jmedchem.9b01304 31746599 10.1021/acs.jmedchem.9b01304

[41] Ase AR, Honson NS, Zaghdane H, Pfeifer TA, Séguéla P (2015) Identification and characterization of a selective allosteric antagonist of human P2X4 receptor channels. Mol Pharmacol 87:606–616. 10.1124/mol.114.096222 25597706 10.1124/mol.114.096222

[42] Mahmood A, Villinger A, Iqbal J (2022) Therapeutic potentials and structure-activity relationship of 1,3-benzodioxole N-carbamothioyl carboxamide derivatives as selective and potent antagonists of P2X4 and P2X7 receptors. Eur J Med Chem 238:114491. 10.1016/j.ejmech.2022.114491 35660250 10.1016/j.ejmech.2022.114491

[43] Inoue K (2021) Nociceptive signaling of P2X receptors in chronic pain states. Purinergic Signal 17:41–47. 10.1007/s11302-020-09743-w 33015745 10.1007/s11302-020-09743-w PMC7955020

[44] D’Antongiovanni V, Pellegrini C, Benvenuti L, Fornai M, Di Salvo C, Natale G, Ryskalin L, Bertani L, Lucarini E, Di Cesare Mannelli L, Ghelardini C, Nemeth ZH, Haskó G, Antonioli L (2022) Anti-inflammatory effects of novel P2X4 receptor antagonists, NC-2600 and NP-1815-PX, in a murine model of colitis. Inflammation 45:1829–1847. 10.1007/s10753-022-01663-8 35338432 10.1007/s10753-022-01663-8 PMC9197920

[45] Namovic MT, Jarvis MF, Donnelly-Roberts D (2012) High throughput functional assays for P2X receptors. Curr Protoc Pharmacol Chap 9:Unit 9.15. 10.1002/0471141755.ph0915s57 10.1002/0471141755.ph0915s57 22684723

[46] Niforatos W, Jarvis MF (2004) Electrophysiological characterization of recombinant and native P2X receptors. Curr Protoc Pharmacol Chap 11:Unit 11.9. 10.1002/0471141755.ph1109s26 10.1002/0471141755.ph1109s26 22294117

[47] Jarvis MF, Bianchi B, Uchic JT, Cartmell J, Lee C-H, Williams M, Faltynek C (2004) [^3^H]A-317491, a novel high-affinity non-nucleotide antagonist that specifically labels human P2X2/3 and P2X3 receptors. J Pharmacol Exp Ther 310:407–416. 10.1124/jpet.103.064907 15024037 10.1124/jpet.103.064907

[48] Michel AD, Miller KJ, Lundström K, Buell GN, Humphrey PP (1997) Radiolabeling of the rat P2X4 purinoceptor: evidence for allosteric interactions of purinoceptor antagonists and monovalent cations with P2X purinoceptors. Mol Pharmacol 51:524–532 9058609

[49] Abdelrahman A, Namasivayam V, Hinz S, Schiedel AC, Köse M, Burton M, El-Tayeb A, Gillard M, Bajorath J, de Ryck M, Müller CE (2017) Characterization of P2X4 receptor agonists and antagonists by calcium influx and radioligand binding studies. Biochem Pharmacol 125:41–54. 10.1016/j.bcp.2016.11.016 27867013 10.1016/j.bcp.2016.11.016

[50] Jin H, Han J, Resing D, Liu H, Yue X, Miller RL, Schoch KM, Miller TM, Perlmutter JS, Egan TM, Tu Z (2018) Synthesis and in vitro characterization of a P2X7 radioligand [^123^I]TZ6019 and its response to neuroinflammation in a mouse model of Alzheimer disease. Eur J Pharmacol 820:8–17. 10.1016/j.ejphar.2017.12.006 29225193 10.1016/j.ejphar.2017.12.006 PMC5767129

[51] Wang M, Gao M, Meyer JA, Peters JS, Zarrinmayeh H, Territo PR, Hutchins GD, Zheng Q-H (2017) Synthesis and preliminary biological evaluation of radiolabeled 5-BDBD analogs as new candidate PET radioligands for P2X4 receptor. Bioorg Med Chem 25:3835–3844. 10.1016/j.bmc.2017.05.031 28554730 10.1016/j.bmc.2017.05.031

[52] Burnstock G (2020) Introduction to purinergic signaling. Methods Mol Bio (Clifton N J) 2041:1–15. 10.1007/978-1-4939-9717-6_1 10.1007/978-1-4939-9717-6_1 31646477

[53] Burnstock G, Boeynaems J-M (2014) Purinergic signalling and immune cells. Purinergic Signal 10:529–564. 10.1007/s11302-014-9427-2 25352330 10.1007/s11302-014-9427-2 PMC4272370

[54] Kiaie SH, Hatami Z, Nasr MS, Pazooki P, Hemmati S, Baradaran B, Valizadeh H (2023) Pharmacological interaction and immune response of purinergic receptors in therapeutic modulation. Purinergic Signal. 10.1007/s11302-023-09966-7 37843749 10.1007/s11302-023-09966-7 PMC11303644

[55] Burnstock G (2016) P2X ion channel receptors and inflammation. Purinergic Signal 12:59–67. 10.1007/s11302-015-9493-0 26739702 10.1007/s11302-015-9493-0 PMC4749528

[56] Dicpinigaitis PV, McGarvey LP, Canning BJ (2020) P2X3-receptor antagonists as potential antitussives: summary of current clinical trials in chronic cough. Lung 198:609–616. 10.1007/s00408-020-00377-8 32661659 10.1007/s00408-020-00377-8

[57] Markham A (2022) Gefapixant: first approval. Drugs 82:691–695. 10.1007/s40265-022-01700-8 35347635 10.1007/s40265-022-01700-8

[58] Inoue K (2022) The role of ATP receptors in pain signaling. Neurochem Res 47:2454–2468. 10.1007/s11064-021-03516-6 35094248 10.1007/s11064-021-03516-6

[59] Domercq M, Matute C (2019) Targeting P2X4 and P2X7 receptors in multiple sclerosis. Curr Opin Pharmacol 47:119–125. 10.1016/j.coph.2019.03.010 31015145 10.1016/j.coph.2019.03.010

[60] Mansoor SE, Lü W, Oosterheert W, Shekhar M, Tajkhorshid E, Gouaux E (2016) X-ray structures define human P2X3 receptor gating cycle and antagonist action. Nature 538:66–71. 10.1038/nature19367 27626375 10.1038/nature19367 PMC5161641

[61] Karasawa A, Kawate T (2016) Structural basis for subtype-specific inhibition of the P2X7 receptor. Elife 5:e22153. 10.7554/eLife.22153 27935479 10.7554/eLife.22153 PMC5176352

[62] Weinhausen S, Nagel J, Namasivayam V, Spanier C, Abdelrahman A, Hanck T, Hausmann R, Müller CE (2022) Extracellular binding sites of positive and negative allosteric P2X4 receptor modulators. Life Sci 311:121143. 10.1016/j.lfs.2022.121143 10.1016/j.lfs.2022.121143 36328074

[63] Markowitz D, Hesdorffer C, Ward M, Goff S, Bank A (1990) Retroviral gene transfer using safe and efficient packaging cell lines. Ann NY Acad Sci 612:407–414. 10.1111/j.1749-6632.1990.tb24328.x 2291567 10.1111/j.1749-6632.1990.tb24328.x

[64] Bradford MMA (1976) rapid and sensitive method for the quantitation of microgram quantities of protein utilizing the principle of protein-dye binding. Anal Biochem 72:248–254. 10.1006/abio.1976.9999 10.1016/0003-2697(76)90527-3 942051

