# Correction to: Preparation and preliminary evaluation of a tritium-labeled allosteric P2X4 receptor antagonist

**DOI:** 10.1007/s11302-024-10030-1

**Published:** 2024-06-19

**Authors:** Jessica Nagel, Olli Törmäkangas, Katja Kuokkanen, Ali El-Tayeb, Josef Messinger, Aliaa Abdelrahman, Christiane Bous, Anke C. Schiedel, Christa E. Müller

**Affiliations:** 1 PharmaCenter Bonn, Pharmaceutical Institute, University of Bonn, Pharmaceutical & Medicinal Chemistry, An der Immenburg 4, 53121 Bonn, Germany; 2 Orion Pharma, Orion Corporation, Tengströminkatu 8, FI-20360 Turku, and Orionintie 1A, 02200 Espoo, FI Finland

**Correction to: Purinergic Signalling**

10.1007/s11302-024-10005-2

The original version of the article unfortunately contained an error.

The second Supplementary Material 2 (word document) has to be removed.

The original article has been corrected.

