# Supplementary material for: Preparation and preliminary evaluation of a tritium-labeled allosteric P2X4 receptor antagonist
Source: Purinergic Signal. 2024 May 25;20(6):645–56. doi: 10.1007/s11302-024-10005-2 (PMC11555173; doi:10.1007/s11302-024-10005-2)
Supplement: Supplementary file 1 — Supplementary Material 1 [file 11302_2024_10005_MOESM1_ESM.pdf]

Supporting Information

Preparation and preliminary evaluation of a tritium-labeled allosteric P2X4 receptor antagonist

Jessica Nagel,[1] Olli Törmäkangas,[2] Katja Kuokkanen,[2] Ali El-Tayeb,[1] Josef Messinger,[2] Aliaa Abdelrahman[1], Christiane Bous[1], Anke C. Schiedel[1] and Christa E. Müller[1*]

[1]University of Bonn, PharmaCenter Bonn, Pharmaceutical Institute, Pharmaceutical & Medicinal Chemistry, An der Immenburg 4, 53121 Bonn, Germany

[2]Orion Pharma, Orion Corporation, Tengströminkatu 8, FI-20360 Turku, and Oriontie 1A, FI-02200 Espoo, Finland

*Author to whom correspondence should be addressed; Email: christa.mueller@uni-bonn.de, Phone: +49-228-73-2301; ORCID: orcid.org/0000-0002-0013-6624

Table of contents

Contents **Page**

Figure S1. Product specification sheet of [^{3}H]PSB-OR-2020. S3

Figure S2. Ultra-high performance liquid chromatogram of [^{3}H]PSB-OR-2020. S4-S5

CAUTION-RADIOACTIVE MATERIAL

Product Specification

Pharmaron UK Ltd
The Old Glassworks
Nettlefold Road, Cardiff, CF24 5JQ
Telephone: +44(0)2920 474900

[^{3}H]Orion Compound
TRQ42131
1 mCi, 37 MBq

Before using this product, please read the instructions on the use, safe handling, storage and disposal of the material.

Technical Data

Specific Activity
Determined by:
Mass Spectrometry : 45 Ci/mmol 1.67 TBq/mmol

Molecular weight (at this specific activity) : 441.7

Date of analysis : 02 April 2020

Radiochemical concentration : 1 mCi/mL 37 MBq/mL

Radiochemical purity by ultra high performance liquid chromatography : 97.7%

Column: Acquity UPLC BEH C18 1.7 µm 50 x 2.1 mm
Solvent A: 15 mM Potassium dihydrogen phosphate in water pH
Solvent B: 2.2 Acetonitrile
Gradient T(min): 0 0.5 10 12 12.5 15
%B: 5 5 85 85 5 5
Temperature: 40°C
Flow rate: 0.9mL/min
Detection: Homogeneous radiochemical detection, UV at 210nm

Chemical Identity
The material co-chromatographs with client supplied material in the above chromatographic system. The mass spectrum is consistent with the proposed structure and a non labelled reference.

Packaging and storage
The material is supplied as a solution in ethanol in a borosilicate multidose vial. Storage at -20°C is recommended.

Preparation
Manufactured to Pharmaron UK Ltd procedures.
[^{3}H]Orion Compound was made from tritium gas by a method developed by Pharmaron UK Ltd. The material was purified by HPLC.

Figure S1. Product specification sheet of [^{3}H]PSB-OR-2020.

康龙化成
PHARMARON

Ultra-High Performance Liquid Chromatogram

TRQ42131 [3H]Orion Compound

Injection date/time: 02/Apr/2020 9:58:45 AM Injection volume: 2.0 μL

Sample ID: Active
Column: Acquity UPLC BEH C18 1.7 μm 50 x 2.1 mm
Column temperature: 40 °C
Eluent A: 15 mM Potassium dihydrogen phosphate in water pH 2.2
Eluent B: Acetonitrile
Gradient T (min) / %B: 0/5, 0.5/5, 10/85, 12/85, 12.5/5, 15/5 @ 0.9 mL/min

Radiochemical Signal

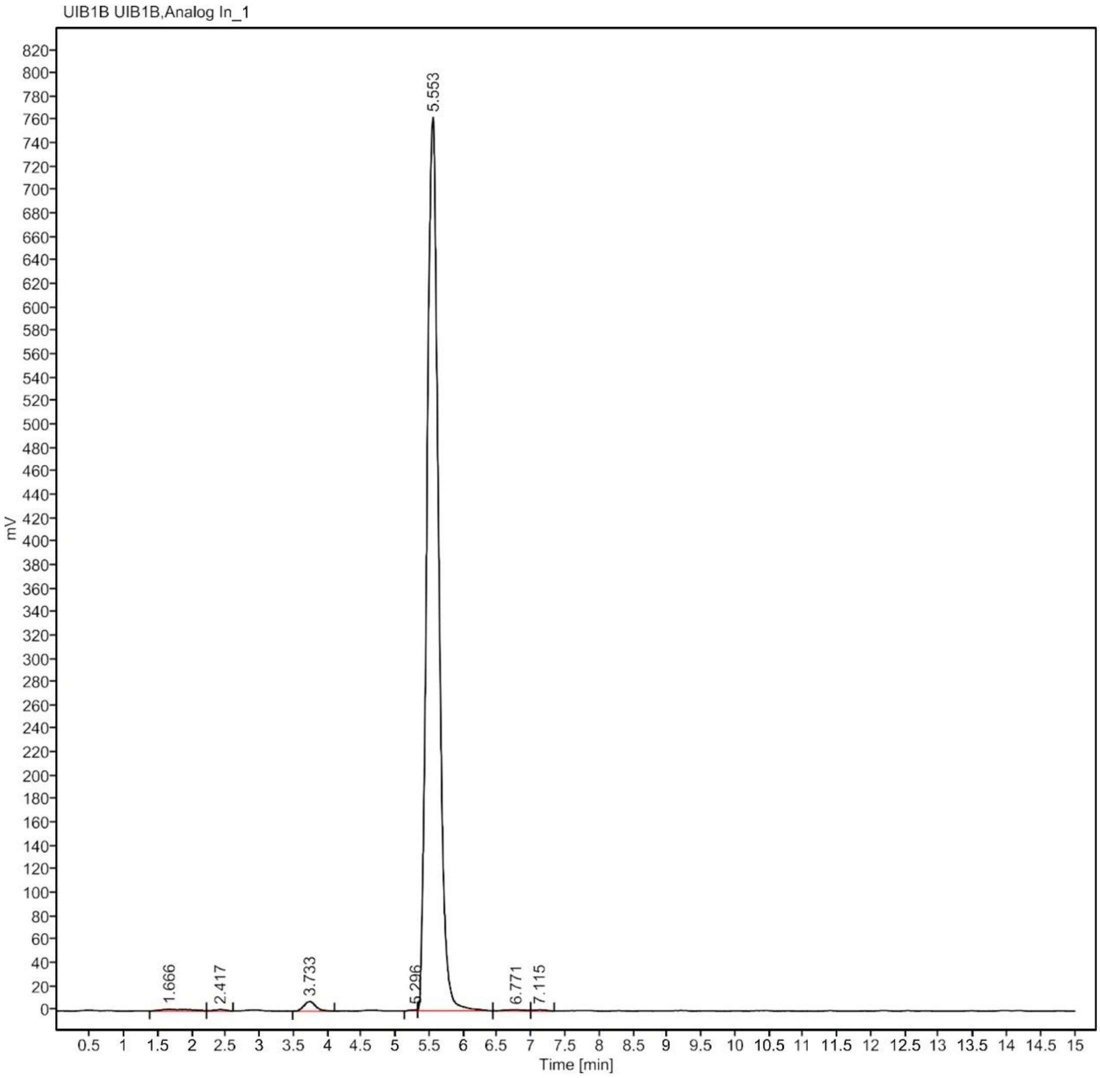

Figure S2. Ultra-high performance liquid chromatogram of [^{3}H]PSB-OR-2020) (page 1).

康龙化成
PHARMARON

Ultra-High Performance Liquid Chromatogram

Signal: UIB1B UIB1B,Analog In_1

Name	Retention Time	Area	Area%
	1.666	49.5444	0.5
	2.417	18.0053	0.2
	3.733	100.3828	1.1
	5.296	7.5553	0.1
	5.553	9118.9207	97.7
	6.771	22.9159	0.2
	7.115	13.2217	0.1
	Sum	9330.5461	

Data file: /QC/Tritium_QC/TRQ42100 to TRQ42199/TRQ42131/Results/H3/TRQ42131_H3.rslt\TRQ42131-H3-1A_200402.dx

Acq. method: TRQ42131_RCP.amx

Result set: TRQ42131_H3

Acq. operator: Lisa Edwards

Report printed by Lisa Edwards

Figure S2. Ultra-high performance liquid chromatogram of [^{3}H]PSB-OR-2020) (page 2).
